# AI ethics in Indian healthcare: a scoping review of national and international guidelines on privacy, data protection, and security

**DOI:** 10.1186/s12910-026-01435-1

**Published:** 2026-03-17

**Authors:** Urvish Joshi, Sharon Baisil, Sanjay Kini B, Margeyi Mehta, Sumit Datta

**Affiliations:** 1 Department of Community Medicine, Narendra Modi Medical College, Gujarat University, Ahmedabad, Gujarat India; 2 Department of Community Medicine, Malankara Orthodox Syrian Church Medical College, Kerala University of Health Sciences, Kolenchery, Kerala India; 3 Department of Community Medicine, Kasturba Medical College, Manipal Academy of Higher Education, Manipal, Karnataka India; 4 Department of Clinical Biochemistry, Medical College, Baroda, The Maharaja Sayajirao University of Baroda, Vadodara, Gujarat India

**Keywords:** Artificial Intelligence, Data Privacy, Medical Ethics, Public Health Policy, Healthcare Governance, Scoping Review

## Abstract

**Objective:**

To systematically map and critically analyze the provisions within Indian national frameworks (Digital Personal Data Protection Act 2023, ICMR guidelines) and key international ethical AI guidelines that address privacy and data security for AI-driven diagnostic tools, specifically within the Indian public health context.

**Methods:**

A scoping review was conducted following the PRISMA-ScR framework. A comprehensive search of government publications, intergovernmental organization reports, and academic literature was performed to identify relevant national and international guidelines. Data were charted and synthesized thematically.

**Results:**

The analysis reveals a fragmented Indian governance landscape, characterized by a principles-based national AI strategy (#AIforAll), sector-specific ethical guidelines (ICMR), and a new, general-purpose data protection law (DPDP Act). While the DPDP Act establishes foundational data fiduciary obligations, its broad exemptions for public health and research create ambiguities for data-intensive AI. International frameworks serve as comparative reference points; the EU AI Act, in particular, offers a contrasting granular, risk-based regulatory model. Key tensions emerge between the universalist principles of international guidelines and the need for a “situated ethics” approach that addresses India’s unique challenges, including the digital divide, data quality issues, and the difficulty of operationalizing meaningful consent.

**Conclusion:**

Existing guidelines provide a foundational but incomplete framework for the safe and ethical deployment of AI diagnostics in Indian public health. There is a critical need to bridge the gap between high-level principles and on-the-ground implementation. This requires developing sector-specific regulations under the DPDP Act, establishing robust standards for data and algorithmic audits, and fostering a cooperative federalism approach that harmonizes international standards with the socio-technical and constitutional realities of India.

**Supplementary Information:**

The online version contains supplementary material available at 10.1186/s12910-026-01435-1.

## Introduction

### Conceptual definitions

To ensure clarity, this review distinguishes between three related but distinct concepts. Privacy is defined here as the fundamental right of individuals to control access to their personal sphere and information. Data Protection refers to the legal frameworks and mechanisms (such as the DPDP Act) designed to safeguard this right. Data Security denotes the technical and organizational measures (e.g., encryption, access controls) implemented to protect data from unauthorized access, manipulation, or loss.

### Rationale

The integration of Artificial Intelligence (AI) into healthcare promises a paradigm shift, particularly for nations like India grappling with immense population scale and systemic challenges. AI-driven diagnostic tools hold the potential to revolutionize public health delivery by addressing critical gaps in access, affordability, and the availability of specialized medical expertise [1]. Early applications demonstrate this promise vividly, with AI models enhancing the speed and accuracy of tuberculosis screening from chest X-rays, detecting diabetic retinopathy to prevent blindness, and improving cancer screening outcomes [2]. These technologies offer to augment scarce human resources and extend the reach of quality diagnostics to underserved rural and remote populations, aligning with national health objectives [3]. 

However, this transformative potential is shadowed by profound ethical, privacy, and data security risks. The very engine of AI—vast quantities of data—becomes a source of vulnerability when the data pertains to sensitive personal health information. These risks are acutely amplified within the unique socio-technical context of India, a nation characterized by a stark digital divide, persistent infrastructural deficits, highly variable digital and health literacy rates, and a deep-seated historical mistrust in public health institutions among certain communities [4]. The deployment of data-hungry AI systems in such an environment raises critical questions about equity, fairness, and the potential for harm.

The central challenge lies at the intersection of technological ambition and regulatory preparedness. India is actively fostering AI innovation through national initiatives like NITI Aayog’s ‘#AIforAll’ strategy, which envisions India as a global hub for AI development [2]. Concurrently, the country has enacted a new, overarching data protection framework, the Digital Personal Data Protection (DPDP) Act, 2023 [5]. This confluence creates a complex and potentially perilous landscape where the rapid adoption of AI technologies may outpace the development of robust, context-aware ethical and legal governance structures.

This situation highlights a deeper, more fundamental tension explored in contemporary academic discourse: the applicability of largely Western-derived, universalist ethical frameworks to the distinct realities of the Global South [6]. Principles of individual autonomy and consent, which are cornerstones of frameworks like the European Union’s General Data Protection Regulation (GDPR) and its successor, the AI Act, may not translate seamlessly to a context where public health decisions are often community-oriented and where obtaining “free, specific, informed, and unambiguous” consent faces significant practical barriers [5]. This has led to calls for a “situated ethics” approach—one that is grounded in the local socio-cultural and infrastructural realities of a nation like India [7]. This scoping review, therefore, seeks to explore how India’s nascent governance frameworks attempt—or fail—to navigate this complex terrain, balancing the drive for innovation with the imperative to protect its citizens.

### Objective

The primary objective of this scoping review is to systematically map, compare, and critically analyze the provisions within existing Indian national guidelines and key international ethical AI frameworks. The analysis focuses specifically on how these documents address the unique privacy and data security implications that arise from the implementation of AI-driven diagnostic tools within the Indian public health sector.

### Research question and framework

This review is guided by the following research question and structured using the PICo framework to ensure clarity and focus.

#### Research question

“How do existing national (Indian, DPDP act and others) and international ethical AI guidelines for healthcare address the unique privacy and data security implications presented by the implementation of AI-driven diagnostic tools in the Indian public health context?”

#### PICo Framework

- Population (P): The Indian public health system, encompassing its diverse stakeholders: patients (as Data Principals), healthcare providers, public health institutions (as Data Fiduciaries), technology developers, and policymakers.
- Interest (I): The specific provisions, principles, and legal requirements pertaining to privacy, data security, informed consent, accountability, liability, and fairness as articulated within ethical and legal guidelines that govern AI-driven diagnostic tools.
- Context (Co): The unique and complex socio-technical, legal, and infrastructural landscape of India, characterized by its federal governance structure, immense demographic and cultural diversity, significant digital divide, and the emerging nature of its data governance regime.

## Methods

### Protocol and registration

This scoping review was conducted in accordance with the methodological framework provided by the PRISMA Extension for Scoping Reviews (PRISMA-ScR) checklist. A formal protocol was registered in a public repository OSF with DOI 10.17605/OSF.IO/V6SY7 prior to the commencement.

### Eligibility criteria

Sources of evidence were included in this review if they met the following criteria:

- Were official national laws, regulations, or policy documents issued by the Government of India pertaining to data protection or national AI strategy;
- Were official ethical guidelines for AI in healthcare or biomedical research published by the Indian Council of Medical Research (ICMR);
- Were official guidelines, recommendations, or legally binding regulations from major international or intergovernmental bodies with significant influence on global AI governance, including the World Health Organization (WHO), Organisation for Economic Co-operation and Development (OECD), UNESCO, the European Union, the United States, and the United Kingdom; and.
- Were published or enacted between 2018 and the present, ensuring relevance to the contemporary landscape of AI development and regulation. Documents were excluded if they were purely commercial in nature, not publicly accessible, or not available in the English language.

### Information sources

A comprehensive search for relevant documents was conducted across a range of authoritative sources. These included official websites of Indian governmental and quasi-governmental bodies such as the Ministry of Electronics and Information Technology (MeitY), NITI Aayog, and the Indian Council of Medical Research (ICMR). International sources included the official portals of the World Health Organization (WHO), the Organisation for Economic Co-operation and Development (OECD), UNESCO, and the European Commission. To capture relevant academic and grey literature, searches were also performed in scholarly databases like PubMed and Scopus.

### Selection of sources of evidence

A two-stage screening process was notionally employed to select the final sources for inclusion. In the first stage, the titles and abstracts (or executive summaries for policy reports and legal documents) of all identified records were screened for their relevance to the research question by authors UJ, SB & MM. Documents that appeared to meet the eligibility criteria proceeded to the subsequent stage, which involved a full-text review to confirm their suitability for inclusion in the final synthesis. Conflict in the said process was resolved by fourth and fifth reviewers, SK and SD.

### Data charting process

A standardized data charting form was developed and utilized to systematically extract pertinent information from each of the selected sources of evidence. This structured approach ensured consistency in data collection across the diverse range of documents. Triple data extraction (by UJ, SB & MM) was followed which was later on compared, duplicity was removed, conflicts were resolved by fourth and fifth reviewers, SK and SD.

### Data items

The variables extracted from each document were organized to facilitate a comprehensive analysis. These included: the issuing body (e.g., Government of India, WHO), the official title of the document, the year of publication or enactment, its legal status (legally binding or non-binding/guideline), its primary geographic scope (national/international), its articulated core ethical principles, and its specific provisions related to the key themes of this review: Data Privacy, Data Security, Consent, Accountability & Liability, Transparency & Explainability, Bias & Fairness, Human Oversight, and Data Governance.

### Critical appraisal of individual sources of evidence

In line with established scoping review methodology, which aims to map the extent and nature of evidence rather than assess its quality, a formal critical appraisal of the individual sources of evidence was not conducted.

### Synthesis of results

The data extracted from the included sources were synthesized using a narrative approach. The findings are structured thematically, organized around the key national and international governance frameworks. To illuminate the relationships between these frameworks, a comparative analysis is presented throughout the narrative and is further consolidated in tabular formats. International frameworks were utilized not as absolute benchmarks, but as comparative reference points to contextualize the Indian approach. This synthesis highlights convergences in principles, divergences in regulatory philosophy, and critical gaps in the existing regulatory landscape.

## Results

### Selection of sources of evidence

**Fig. 1:**
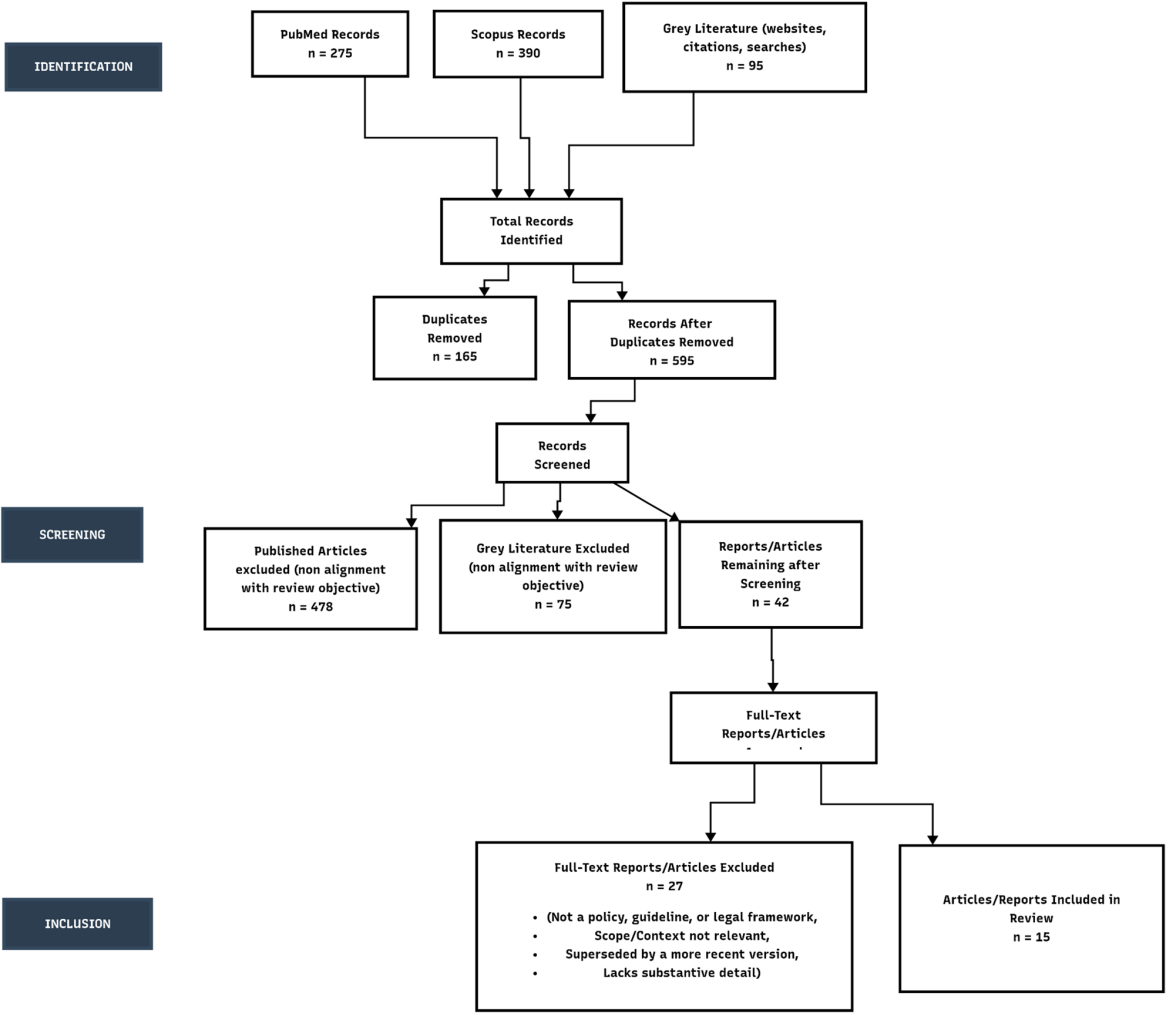
PRISMA-ScR flow diagram for screening and selection of records

The selection process identified a core set of foundational documents from Indian national bodies and key international organizations. After screening, a total of 15 primary legal, policy, and ethical guidance documents were included for detailed analysis. These documents form the basis of this review. (Figure 1).

### Characteristics of sources of evidence

The included sources represent a spectrum of governance instruments. At the national level for India, this includes legally binding, cross-sectoral legislation (the Digital Personal Data Protection Act, 2023), non-binding but influential national strategy documents (NITI Aayog’s National Strategy for Artificial Intelligence), and sector-specific, non-binding ethical guidelines (ICMR’s Ethical Guidelines for AI in Healthcare, 2023). The international sources are similarly diverse, comprising influential non-binding principles from intergovernmental organizations (WHO, OECD, UNESCO) and the pioneering legally binding, risk-based regulation from the European Union (the EU AI Act). This heterogeneity in legal status and scope is central to understanding the current state of AI governance.

### Results of individual sources of evidence & comparative analysis

#### The Indian governance framework: a tripartite structure

India’s approach to governing AI in healthcare is not monolithic but rather a composite of three distinct pillars: a foundational data protection law, an aspirational innovation strategy, and sector-specific ethical guidance.

A. The Digital Personal Data Protection (DPDP) Act, 2023: The Legal Foundation

The DPDP Act, 2023, represents India’s first comprehensive legislation dedicated to data privacy, establishing a principles-based framework for the processing of *digital personal data* [5]. It introduces and defines key roles and responsibilities, designating the individual whose data is processed as the ‘Data Principal’ and the entity that determines the purpose and means of processing as the ‘Data Fiduciary’ [7]. The Act stipulates that all data processing must be for a “lawful purpose” and grounded in one of two legal bases: the explicit consent of the Data Principal or “certain legitimate uses” specified within the Act [6]. 

On the crucial issues of privacy and consent, the DPDP Act sets a high bar. Consent must be “free, specific, informed, unconditional, and unambiguous with a clear affirmative action” [8]. This requires Data Fiduciaries to provide a clear, itemized notice to the Data Principal before or at the time of requesting consent, detailing the data to be collected and the specific purpose of processing. The Act also envisions a system of ‘Consent Managers’—registered entities that will act as a single point of contact to help individuals manage, review, and withdraw their consent across various platforms [9]. 

Regarding security and accountability, the Act mandates that Data Fiduciaries implement “appropriate technical and organisational measures” to protect personal data and prevent breaches [10]. In the event of a breach, the Fiduciary is obligated to notify both the newly established Data Protection Board of India and each affected Data Principal [3]. The Act introduces the concept of a ‘Significant Data Fiduciary’ (SDF), a designation for entities that handle large volumes or highly sensitive types of personal data. SDFs are subject to more stringent obligations, including the mandatory appointment of a Data Protection Officer (DPO) based in India, the commissioning of independent data auditors, and the undertaking of periodic Data Protection Impact Assessments (DPIAs) [2]. 

A critical feature of the DPDP Act for the healthcare sector is Sect. 7, which outlines “certain legitimate uses” for which personal data can be processed *without* obtaining explicit consent from the Data Principal [11]. These uses include responding to a “medical emergency involving a threat to the life or immediate threat to the health” of an individual and for measures related to “medical treatment or health services” during an epidemic or other threat to public health. While these exemptions are pragmatically necessary for public health operations, they create a significant area of ambiguity when applied to the development of data-intensive AI systems [12]. The processing of millions of patient records to train a diagnostic AI for a prevalent disease like tuberculosis could arguably be framed as a measure to manage a public health threat, potentially allowing public health authorities to bypass the Act’s stringent individual consent requirements. This creates a direct tension between individual data rights and collective public health objectives, a conflict that the Act itself does not explicitly resolve, thereby highlighting a regulatory gap regarding the application of this ‘legitimate use’ gateway.

B. NITI Aayog's National Strategy for AI: The Aspirational Vision

NITI Aayog’s 2018 discussion paper, “National Strategy for Artificial Intelligence,” lays out an aspirational vision for India’s AI future, centered on the philosophy of ‘#AIforAll’ [13]. This strategy aims to leverage AI as a catalyst for inclusive social and economic growth, identifying healthcare as a primary sector for intervention [2]. The core goal is to use AI to tackle India’s long-standing healthcare challenges of quality, accessibility, and affordability [7]. 

The strategy recognizes that data is the lifeblood of AI and identifies concerns around data privacy and security as major barriers to adoption. To address this and spur innovation, it proposes the creation of multi-stakeholder “data marketplaces” and the development of large foundational datasets. The intent is to formalize the data exchange economy, lower entry barriers for startups, and promote “data democratization” [13]. 

From an ethical standpoint, the document references the common FAT (Fairness, Accountability, and Transparency) framework and suggests the establishment of a consortium of Ethics Councils at research centers [14]. However, the strategy itself is high-level and does not prescribe a detailed ethical or privacy framework, largely deferring to other regulatory bodies and future legislation. This pro-innovation stance, which includes potential exemptions for startups, creates an inherent tension with the cautious, rights-based approach required for robust data protection [15]. The focus on creating a vibrant supply of data and AI tools through marketplaces, while beneficial for the tech ecosystem, raises profound privacy questions in the healthcare context: How is data anonymized to a sufficient standard? Who holds ownership of data within these marketplaces? And how are patients, the ultimate source of the data, compensated or given a voice in its use? The strategy prioritizes the “how-to” of building an AI ecosystem, while deferring specific ethical and protective governance mechanisms to other regulatory bodies.

C. ICMR's Ethical Guidelines for AI in Healthcare (2023): The Sector-Specific Moral Compass

The “Ethical Guidelines for Application of Artificial Intelligence in Biomedical Research and Healthcare,” released by the Indian Council of Medical Research (ICMR) in 2023, represents the most direct and contextually relevant framework for this review [15]. These guidelines are explicitly designed to supplement India’s broader biomedical research ethics for the unique challenges posed by AI. They are built upon ten core, patient-centric ethical principles: Autonomy, Safety and Risk Minimization, Trustworthiness, Data Privacy, Accountability and Liability, Optimization of Data Quality, Accessibility, Equity and Inclusiveness, Collaboration, and Validity [16]. 

The ICMR guidelines provide granular, health-specific interpretations of broader legal requirements. Where the DPDP Act mandates “appropriate security measures,” the ICMR specifies this should include robust encryption, data anonymization wherever possible, strict access controls, and privacy impact assessments [17]. Where the DPDP Act requires consent, the ICMR details what *informed consent* means in the context of AI research: clearly explaining the respective roles of human clinicians and technology, the potential risks of algorithmic errors, and the patient’s unequivocal right to opt-out of AI-driven interventions [15]. 

Crucially, the guidelines champion the “Human in The Loop” (HITL) model, asserting that humans must retain ultimate oversight and control over clinical decision-making [18]. On accountability, they propose a shared liability model where developers and manufacturers may be held responsible for harm caused by algorithmic flaws, while healthcare professionals and institutions are accountable for its clinical deployment and use. The guidelines also identify data bias as the “greatest threat” to data-driven AI in health [15]. They call for training datasets to be meticulously curated to be representative of India’s diverse population, including marginalized and remote communities, and for independent audits to proactively identify and mitigate biases.

The ICMR guidelines are thus an essential instrument, attempting to bridge the gap between the high-level legal principles of the DPDP Act and the practical realities of AI deployment in healthcare. However, their primary limitation is their legal status. As ethical guidelines, their enforcement depends on the diligence and capacity of institutional Ethics Committees (ECs) [18]. While influential, they do not carry the same force as hard law, creating potential inconsistencies in application and oversight, especially in large-scale public health initiatives that may operate outside traditional research settings.

### Key international governance frameworks: a comparative overview

The global discourse on AI ethics has produced several influential frameworks that provide a crucial benchmark against which to evaluate India’s approach.

A. World Health Organization (WHO): A Public Health & Equity Focus

The WHO’s guidance on AI for health is anchored in a public health and equity perspective. It articulates six core principles:

1. protecting human autonomy;
2. promoting human well-being, safety, and the public interest;
3. ensuring transparency, explainability, and intelligibility;
4. fostering responsibility and accountability;
5. ensuring inclusiveness and equity; and.
6. promoting responsive and sustainable AI. [19].

A central tenet of the WHO’s position is that AI must not exacerbate existing health disparities or deepen the digital divide; instead, it should be a tool for achieving universal health coverage [20]. The guidance strongly advocates for human oversight in clinical decision-making and the establishment of clear accountability frameworks. More recent guidance on Large Multimodal Models (LMMs) specifically flags the risks of factual inaccuracies, embedded data biases, and the challenges of securing meaningful informed consent in patient-facing applications [21].

B. Organisation for Economic Co-operation and Development (OECD): A Focus on Trustworthy Innovation

The OECD AI Principles aim to foster an environment conducive to “trustworthy AI” that balances innovation with robust safeguards. The framework is built on five values-based principles:

1. inclusive growth, sustainable development, and well-being;
2. human-centered values and fairness;
3. transparency and explainability;
4. robustness, security, and safety; and
5. accountability.

The OECD’s approach emphasizes the importance of a systematic, lifecycle-based risk management process and encourages international cooperation, public-private partnerships, and continued investment in AI research and development to address technical and ethical challenges [22].

C. UNESCO: A Human Rights-Based Approach

UNESCO’s “Recommendation on the Ethics of Artificial Intelligence” adopts a comprehensive, multicultural, and human rights-centric perspective. It is founded on four core values—respect for human rights, flourishing of ecosystems, ensuring diversity, and living in peaceful societies—and translated into ten actionable principles, including proportionality, safety and security, privacy and data protection, human oversight, and fairness [23]. Significantly, the UNESCO recommendation explicitly acknowledges the risk of ethical and technological divides, calling for specific attention to be paid to the needs and contexts of Low- and Middle-Income Countries (LMICs), which have often been underrepresented in the global AI ethics debate [24]. It warns against the uncritical imposition of dominant value systems and advocates for the inclusion of local knowledge and cultural pluralism.

D. The European Union (EU) AI Act: A Legally Binding, Risk-Based Model

The EU AI Act stands apart as the world’s first comprehensive, legally binding regulation specifically for artificial intelligence. Its defining feature is a risk-based classification system that categorizes AI applications into four tiers: unacceptable risk (which are banned outright), high-risk, limited risk, and minimal risk [25]. AI systems intended for use in medical devices or for critical public service functions—such as determining eligibility for healthcare benefits or performing emergency patient triage—are generally classified as “high-risk” [26]. 

This high-risk designation triggers a suite of stringent legal obligations that must be met *before* the AI system can be placed on the market. These ex-ante requirements include:

- Rigorous Data Governance: The datasets used for training, validating, and testing the AI system must be of high quality, meaning they are relevant, sufficiently representative, and, to the greatest extent possible, free of errors and biases.
- Comprehensive Technical Documentation: Providers must create and maintain detailed technical documentation demonstrating the system's compliance with all requirements of the Act.
- Transparency and User Information: Systems must be designed for transparency, and deployers must be provided with clear, comprehensive instructions for use, outlining the system's capabilities and limitations.
- Mandatory Human Oversight: High-risk systems must be designed and developed in such a way as to enable effective human oversight, allowing a person to intervene, override, or stop the system to prevent or minimize risks.
- *High Standards of Robustness, Accuracy, and Cybersecurity*: Systems must meet demanding technical standards to ensure they are resilient, perform accurately, and are protected against vulnerabilities.

### Comparative synthesis: gaps and convergences

The various frameworks can be understood as existing on a governance spectrum. At one end, UNESCO and the OECD provide high-level, globally applicable principles designed to shape norms and values. The WHO narrows this focus to the specific domain of health, with a strong emphasis on equity. India’s ICMR guidelines offer a more granular ethical code of conduct for the health sector, while the DPDP Act establishes a general legal floor for data protection across all sectors. At the other end of the spectrum, the EU AI Act represents the most prescriptive, legally binding, and risk-stratified model, moving from principles to detailed compliance obligations. While India’s composite framework touches upon elements from each part of this spectrum, it currently lacks the binding legal specificity and ex-ante enforcement mechanisms for high-risk AI that define the EU’s approach.

Tables 1 and 2 provide a structured comparison, first of the core principles and then of the specific privacy and security provisions, to illuminate these convergences and divergences more clearly.

**Table 1:** Comparative Analysis of Core Principles in Key AI Governance Frameworks

Principle	ICMR (India)	WHO	OECD	UNESCO	EU AI Act (High-Risk)
Human Autonomy / Oversight	Patient-centric; “Human in The Loop” (HITL) must have ultimate control over clinical decisions.	Protect human autonomy; humans should remain in control of healthcare systems and medical decisions.	Human-centered values; implement safeguards for human agency and oversight.	Human oversight and determination; ensure ultimate human responsibility and accountability.	Legally mandated requirement; systems must be designed to be effectively overseen by humans.
Safety / Non-maleficence	Paramount importance of patient safety; risk minimization throughout the AI lifecycle.	Promote human well-being and safety; AI should not harm people; requires regulatory approval for safety and accuracy.	Robustness, security, and safety; systems should not pose unreasonable safety risks and should fail safely.	Safety and security; unwanted harms (safety risks) and vulnerabilities (security risks) should be avoided and addressed.	Legally binding requirement; systems must be robust, secure, and safe, with risk management systems in place.
Fairness / Equity	Accessibility, equity, and inclusiveness; use representative data to prevent bias against any group; local language interfaces.	Ensure inclusiveness and equity; AI must not exacerbate health disparities; address the digital divide.	Inclusive growth and fairness; advance inclusion of underrepresented populations and reduce inequalities.	Fairness and non-discrimination; promote social justice and ensure benefits are accessible to all.	Legally mandated requirement; training data must be representative to prevent discriminatory biases.
Transparency / Explainability	Trustworthiness principle includes explainability and transparency; logic should be understandable to providers and patients.	Ensure transparency, explainability, and intelligibility; sufficient information should be documented and published.	Transparency and explainability; provide meaningful, plain-language information about system logic and data sources.	Transparency and explainability; public should be informed when a decision is based on AI; T&E should be appropriate to context.	Legally mandated requirement; systems must be transparent and accompanied by clear instructions for use for deployers.
Accountability / Responsibility	Shared liability model; developers for flaws, clinicians for deployment. Mechanism for redress needed.	Foster responsibility and accountability; clear frameworks needed to define responsibilities of all stakeholders	Accountability; AI actors are accountable for proper functioning and respecting principles based on their roles.	Responsibility and accountability; AI systems should be auditable and traceable with oversight mechanisms.	Legally defined obligations for providers, importers, and deployers; post-market monitoring and incident reporting required.
Privacy / Data Protection	Data privacy is a core principle; ensure protection at all stages via anonymization, encryption, and user control.	Protection of privacy and confidentiality is essential for human autonomy; requires valid informed consent.	Respect for privacy and data protection is a core human-centered value.	Right to privacy and data protection; privacy must be protected throughout the AI lifecycle; adequate frameworks needed.	Aligns with GDPR; data governance is a key requirement for high-risk systems, focusing on data quality and relevance.

This table provides a comparative analysis of core principles in key AI governance frameworks [15–17, 21, 27]

**Table 2:** Mapping of Specific Privacy & Security Provisions for AI Diagnostics

Provision	Indian Framework (DPDP Act + ICMR Guidelines)	EU AI Act (for High-Risk Systems)
Legal Basis for Processing Health Data	Consent (specific, informed, unambiguous) OR “Certain Legitimate Uses” (e.g., public health threat).	Compliance with GDPR; processing of health data requires an explicit legal basis, typically explicit consent or substantial public interest.
Consent Requirements	High standard of consent required by DPDP Act. ICMR adds need to explain AI risks and opt-out rights. Ambiguity due to ‘legitimate use’ exemption.	Governed by GDPR. Consent must be explicit for processing sensitive data. AI Act focuses on transparency to users, not consent from data subjects.
Data Quality & Bias Mitigation	ICMR principle: Data must be representative of Indian population, free from bias. Non-binding ethical guidance.	Legally binding requirement: Training, validation, and testing data must be relevant, representative, free of errors, and complete.
Security Measures	DPDP Act: “Appropriate technical and organisational measures”. ICMR: Recommends encryption, anonymization, access controls.	Legally binding requirement: Systems must achieve an appropriate level of accuracy, robustness, and cybersecurity.
Breach Notification	Mandatory under DPDP Act to notify Data Protection Board and affected Data Principals.	Handled under GDPR. For AI systems, serious incidents must be reported to market surveillance authorities.
Human Oversight Mandates	ICMR ethical principle: “Human in The Loop” is essential. Not a uniform legal mandate across all applications.	Legally binding design requirement: High-risk systems must be designed and developed to be effectively overseen by natural persons.
Liability for Harm	Unclear. DPDP Act is silent on liability for harm. ICMR suggests a shared model (developer/clinician) but it is not legally binding.	EU Product Liability Directive applies; victims can claim compensation from manufacturers for damage caused by defective products, including AI.
Algorithmic Audit Requirements	ICMR recommends audits, and SDFs under DPDP Act must have data auditors. Not a universal pre-market requirement for all health AI.	Legally binding requirement: All high-risk AI systems must undergo a conformity assessment before being placed on the market.

This table provides a mapping of specific privacy & security provisions for AI diagnostics [8, 15, 27]

## Discussion

### Summary of evidence

The evidence synthesized in this review demonstrates that India’s governance framework for AI in healthcare is a multifaceted but fragmented mosaic. It comprises a general-purpose data protection law (DPDP Act), an aspirational, innovation-focused national strategy (NITI Aayog), and a set of domain-specific, non-binding ethical guidelines (ICMR). While these components are philosophically aligned with the core principles of trustworthy AI articulated in major international frameworks—such as those from the WHO, OECD, and UNESCO—they collectively lack the legal and regulatory granularity required to comprehensively address the high-stakes privacy and security risks inherent in AI-driven diagnostics, particularly within the public health system.

In stark contrast, the international landscape is moving towards more integrated and prescriptive models. The EU AI Act, in particular, establishes a benchmark for legally binding, risk-based regulation that imposes stringent, ex-ante obligations on developers and deployers of high-risk AI systems [27]. The comparison reveals that while India has laid a foundational groundwork, critical gaps remain in translating high-level principles into enforceable, context-specific legal requirements for the healthcare AI lifecycle.

### Analysis of critical gaps and tensions

The implementation of AI diagnostic tools in the Indian public health context is fraught with several critical challenges that the current governance frameworks do not fully resolve.

#### The accountability gap

A significant gap exists in establishing a clear, legally enforceable mechanism for accountability and redress in cases of AI-induced harm.

The DPDP Act defines the responsibilities of Data Fiduciaries but is largely silent on liability for harms caused by algorithmic decisions [9]. While existing Indian legal frameworks such as the Consumer Protection Act, 2019, and principles of medical negligence under tort law theoretically cover healthcare harms, their application to ‘black-box’ algorithmic errors remains legally untested. The ICMR guidelines astutely propose a shared liability model, distinguishing between developer responsibility for algorithmic flaws and clinician responsibility for deployment [15]. To operationalize this, legal clarity is required on ‘who pays, who explains, and who fixes’—specifically delineating the financial and legal liability of the developer (for product failure), the hospital (for deployment protocols), and the clinician (for final decision-making). However, this remains an ethical recommendation, not a legal mandate. In the complex ecosystem of Indian public health, which often involves public-private partnerships, determining liability becomes exceedingly difficult. The question of how a patient in a remote primary health center, potentially harmed by a faulty AI diagnosis delivered through a government program using a privately developed tool, can seek effective recourse remains unanswered by the current frameworks [13]. 

#### The data quality and bias paradox

A consensus across all robust frameworks (ICMR, WHO, EU AI Act) is the critical importance of using high-quality, representative data to train AI models to ensure fairness and prevent bias [10, 15, 27]. India faces a fundamental paradox in this regard. The public health system, especially in rural and underserved areas, is often characterized by incomplete, non-standardized, and non-digitized data, reflecting deep-seated societal inequities [28]. To build the unbiased AI tools needed to improve this system, developers must rely on data that is inherently biased and of variable quality. This creates a concerning feedback loop where AI tools, trained on unrepresentative data, could systematically misdiagnose or discriminate against the very populations they are intended to serve. High-level principles calling for fairness are insufficient to break this cycle without concrete, mandated standards for data collection, auditing, and bias mitigation.

#### The Challenge of “Meaningful Consent”

The DPDP Act’s standard for consent—“free, specific, informed, unconditional, and unambiguous”—is a robust ideal that is exceptionally difficult to operationalize in Indian public health settings [8]. Factors such as low health and digital literacy, linguistic diversity, and the inherent power imbalance between a state-run health service and its beneficiaries can render the consent process a mere formality rather than a meaningful exercise of autonomy [9]. This challenge is compounded by the DPDP Act’s broad exemption for “legitimate uses” in public health [5]. This provision could be interpreted as a legal gateway to bypass individual consent for the large-scale data aggregation necessary for training AI models, creating a direct conflict with the ICMR’s strong ethical emphasis on patient autonomy and informed choice [15]. To address these literacy barriers, a shift from lengthy legalistic notices to ‘tiered’ and ‘visual’ consent models in local vernaculars is necessary, ensuring that consent is an ongoing process rather than a one-time checkbox.

#### The Clash of paradigms: universalism vs. situated ethics

International guidelines are predominantly shaped by the experiences and value systems of the Global North, presupposing robust legal infrastructures, high digital literacy, and a strong emphasis on individual rights [29]. The direct transposition of these universalist frameworks onto India risks becoming a form of “ethical colonialism,” failing to account for local realities. As argued in academic literature, a “situated ethics” approach is necessary—one that is co-designed with local communities and is explicitly tailored to address India’s unique challenges, such as its collectivist social fabric, linguistic diversity, and socio-economic disparities [15]. While India’s frameworks, particularly the ICMR guidelines with their call for local language interfaces, show nascent signs of this approach, they remain heavily influenced by the dominant global discourse [29]. 

### Implications for policy and practice in India

To bridge these gaps and foster a truly trustworthy AI ecosystem in healthcare, Indian policymakers and stakeholders should consider the following actions:

1. Harmonize Governance via a Unified Pathway: There is a critical need to bridge the fragmentation between the DPDP Act, NITI Aayog strategies, and ICMR guidelines. A unified 'Health AI Pathway' should be established where compliance with the DPDP Act is the legal baseline, and ICMR ethical clearance is the mandatory sectoral standard. The Central Government should leverage its authority to notify specific rules that clarify the 'legitimate use' exemption, ensuring it is not a loophole but a regulated corridor requiring specific data audits.
2. Legally Mandate AI Risk Management and Audits*:* India should evolve its governance model towards a legally binding, risk-based approach, drawing inspiration from the EU AI Act. This would involve legally mandating risk management practices throughout the AI lifecycle for high-risk diagnostic tools. It should include requirements for pre-deployment conformity assessments by independent auditors and robust post-market surveillance to monitor performance and safety in real-world conditions. The NIST AI Risk Management Framework offers a flexible and comprehensive model that could be adapted for this purpose [30].
3. Establish Indian-Specific Data Standards: There is an urgent need to establish and enforce national standards for health data quality, anonymization, and representativeness. This is not merely a technical task but a crucial step towards equity. It requires developing protocols for proactive data collection from diverse and marginalized populations to ensure that the datasets used to train AI models reflect the true diversity of India, thereby actively mitigating the risk of algorithmic bias.
4. Strengthen the Role of Ethics Committees and the Data Protection Board: The capacity of institutional Ethics Committees must be significantly enhanced through training and resources to enable them to effectively review complex AI research protocols. Furthermore, the forthcoming Data Protection Board of India must be constituted with deep technical expertise, empowering it to audit not just data handling practices but the algorithms themselves, ensuring they are fair, transparent, and safe.
5. Adopt a Cooperative Federalism Approach: A socio-legal analysis reveals a constitutional tension: 'Health' is a State subject (List II), while 'Data Protection' falls under Central jurisdiction. A centralized AI regulator may face enforcement challenges in state-run public health systems. Therefore, a model of 'cooperative federalism' is preferable, where central standards (DPDP) are operationalized through State Health Mission MoUs, allowing for localized adaptation and enforcement.

### Review limitations

This scoping review has several limitations. The analysis is confined to publicly available, English-language documents, which may exclude some regional or non-English policy discussions. The regulatory landscape for AI is evolving with extreme rapidity, rendering this review a snapshot at a specific point in time; new guidelines and rules are expected. Most critically, the practical enforcement of the DPDP Act and the operational functioning of the Data Protection Board are yet to be observed. A significant limitation is the reliance on policy documents and the absence of empirical data, case law, or documented deployment experiences. This limits the analysis to the text of the laws and guidelines, rather than their real-world impact, which will be a crucial area for future research. Furthermore, consistent with scoping review methodology, this study maps the existing textual landscape of governance frameworks but does not evaluate the practical effectiveness, stakeholder compliance, or on-the-ground enforcement of these guidelines.

## Conclusion

India stands at a critical juncture in the adoption of AI for healthcare. The nation has constructed a foundational governance framework through a patchwork of general data protection law, national AI strategy, and sector-specific ethical guidance. However, this review concludes that this patchwork, while philosophically aligned with global principles, contains significant gaps and ambiguities when tested against the unique privacy, security, and ethical challenges posed by AI-driven diagnostics in the Indian public health system.

The fundamental challenge for India is to move beyond high-level principles and translate them into enforceable, context-specific regulations that can function effectively within its complex socio-technical environment. The path forward requires the development of a hybrid governance model. This model must integrate the legal certainty and risk-based rigor exemplified by frameworks like the EU AI Act with the contextual sensitivity and cultural awareness of a “situated ethics” approach, as championed by the ICMR and critical academic voices. Working towards this balance through targeted rulemaking, capacity building, and the establishment of robust auditing standards will be crucial for ensuring that the transformative promise of AI in Indian healthcare can be realized safely, equitably, and in a manner that builds, rather than erodes, the trust of the public it is meant to serve.

## Supplementary Information

Supplementary Material 1.

## Data Availability

All data generated or analysed during this study are included in this published article. The national and international policy documents, guidelines, and legal acts reviewed are publicly accessible through the respective institutional repositories cited in the references.
