# Supplementary material for: AI ethics in Indian healthcare: a scoping review of national and international guidelines on privacy, data protection, and security
Source: BMC Med Ethics. 2026 Mar 17;27:86. doi: 10.1186/s12910-026-01435-1 (PMC13107574; doi:10.1186/s12910-026-01435-1)
Supplement: Supplementary file 1 — Supplementary Material 1. [file 12910_2026_1435_MOESM1_ESM.docx]

## **Database Search Strategies**

####

#### **PubMed Search Strategy (last accessed on July 2, 2025)**

Search: ( ("artificial intelligence"[MeSH Terms] OR "artificial intelligence"[tiab] OR "machine learning"[tiab] OR "deep learning"[tiab] OR "algorithm*"[tiab] OR "predictive analytics"[tiab] OR "digital health"[tiab] OR "health informatics"[tiab]) AND ("diagnostic"[tiab] OR "diagnostics"[tiab] OR "screening"[tiab] OR "medical imaging"[tiab] OR "diagnostic imaging"[MeSH Terms] OR "diagnostic techniques and procedures"[MeSH Terms]) AND ("privacy"[MeSH Terms] OR "privacy"[tiab] OR "data protection"[tiab] OR "data security"[tiab] OR "confidentiality"[tiab] OR "data governance"[tiab] OR "data sharing"[tiab] OR "information security"[tiab]) AND ("ethics"[MeSH Terms] OR "ethics"[tiab] OR "ethical guidelines"[tiab] OR "bioethics"[tiab] OR "governance"[tiab] OR "regulation"[tiab] OR "policy"[tiab] OR "guideline"[tiab] OR "standards"[tiab] OR "transparency"[tiab] OR "accountability"[tiab]) AND ("India"[MeSH Terms] OR "India"[tiab] OR "Indian"[tiab] OR "public health"[tiab] OR "healthcare"[tiab] OR "health care"[tiab] OR "South Asia"[tiab] OR "LMIC"[tiab] OR "low- and middle-income countr*"[tiab]) ) AND ("2018/01/01"[Date - Publication] : "3000"[Date - Publication]) AND (english[lang])

("artificial intelligence"[MeSH Terms] OR "artificial intelligence"[Title/Abstract] OR "machine learning"[Title/Abstract] OR "deep learning"[Title/Abstract] OR "algorithm*"[Title/Abstract] OR "predictive analytics"[Title/Abstract] OR "digital health"[Title/Abstract] OR "health informatics"[Title/Abstract]) AND ("diagnostic"[Title/Abstract] OR "diagnostics"[Title/Abstract] OR "screening"[Title/Abstract] OR "medical imaging"[Title/Abstract] OR "diagnostic imaging"[MeSH Terms] OR "diagnostic techniques and procedures"[MeSH Terms]) AND ("privacy"[MeSH Terms] OR "privacy"[Title/Abstract] OR "data protection"[Title/Abstract] OR "data security"[Title/Abstract] OR "confidentiality"[Title/Abstract] OR "data governance"[Title/Abstract] OR "data sharing"[Title/Abstract] OR "information security"[Title/Abstract]) AND ("ethics"[MeSH Terms] OR "ethics"[Title/Abstract] OR "ethical guidelines"[Title/Abstract] OR "bioethics"[Title/Abstract] OR "governance"[Title/Abstract] OR "regulation"[Title/Abstract] OR "policy"[Title/Abstract] OR "guideline"[Title/Abstract] OR "standards"[Title/Abstract] OR "transparency"[Title/Abstract] OR "accountability"[Title/Abstract]) AND ("India"[MeSH Terms] OR "India"[Title/Abstract] OR "Indian"[Title/Abstract] OR "public health"[Title/Abstract] OR "healthcare"[Title/Abstract] OR "health care"[Title/Abstract] OR "South Asia"[Title/Abstract] OR "LMIC"[Title/Abstract] OR "low and middle income countr*"[Title/Abstract]) AND 2018/01/01:3000/12/31[Date - Publication] AND "english"[Language]

####

#### **Scopus Search Strategy (last accessed on July 2, 2025)**

(

TITLE-ABS-KEY("artificial intelligence" OR "machine learning" OR "deep learning")

AND

TITLE-ABS-KEY("diagnostic" OR "diagnostics" OR "screening" OR "medical imaging")

AND

TITLE-ABS-KEY("privacy" OR "data protection" OR "data security" OR "confidentiality")

AND

TITLE-ABS-KEY("ethics" OR "ethical guidelines" OR "bioethics" OR "governance" OR "regulation")

AND

TITLE-ABS-KEY("India" OR "Indian" OR "public health" OR "healthcare" OR "health care")

)

AND

(PUBYEAR > 2017)

AND

(LIMIT-TO(LANGUAGE, "English"))
